# Association Between Cardiac and Renal Biomarkers in IgA Nephropathy: Insights into Cardiorenal Interaction: A Cross-Sectional Study

**DOI:** 10.3390/biomedicines14081835

**Published:** 2026-08-14

**Authors:** Balázs Sági, Éva Fejes, Rita Klaudia Jakabfi-Csepregi, Kőszegi Tamás, Tibor József Kovács

**Affiliations:** 1 2nd Department of Internal Medicine and Nephrology, Diabetes Center, Clinical Centre, Medical School, University of Pécs, 7624 Pécs, Hungary; kovacs.tibor.jozsef@pte.hu; 2 National Dialysis Center Pécs, 7624 Pécs, Hungary; 3 Hospital of Komló, University of Pécs, 7624 Pécs, Hungary; efchengirl@gmail.com; 4 Institute of Laboratory Medicine, Medical School, University of Pécs, 7624 Pécs, Hungary; ritacsepregi93@gmail.com (R.K.J.-C.); koszegi.tamas@pte.hu (K.T.)

**Keywords:** chronic kidney disease, IgA nephropathy, renal function, heart type-fatty acid binding protein, mid-regional prohormone of atrial natriuretic peptide, galectin-3

## Abstract

**Background**: IgA nephropathy (IgAN) is the most common primary glomerular disease worldwide and is associated with increased cardiovascular (CV) morbidity and mortality. Galectin-3 is a marker of fibrosis and adverse CV outcomes in chronic kidney disease (CKD), while mid-regional pro-atrial natriuretic peptide (MR-proANP) and heart-type fatty acid-binding protein (H-FABP) reflect myocardial wall stress and subclinical myocardial injury. Data on the combined behavior of these biomarkers in IgAN are limited. We investigated their association with renal function and cardiovascular parameters in IgAN. **Methods**: In this cross-sectional study, 90 patients with biopsy-proven IgAN were examined, but 76 patients’ data were evaluated. Serum galectin-3, MR-proANP, and H-FABP concentrations were measured by ELISA. Renal function was assessed using estimated glomerular filtration rate (eGFR, CKD-EPI). All patients underwent transthoracic echocardiography and arterial stiffness assessment, including pulse wave velocity of the aorta (PWVao) and central systolic blood pressure (SBPao). **Results**: Patients with reduced renal function (eGFR <60 mL/min/1.73 m^2^) had significantly higher galectin-3 (*p* = 0.011), MR-proANP (*p* < 0.001), and H-FABP (*p* < 0.001) concentrations. Biomarker levels increased progressively with declining eGFR. Galectin-3 concentrations were higher in patients with increased left ventricular mass index (*p* = 0.015). All three biomarkers were elevated in patients with higher central aortic systolic pressure. MR-proANP and H-FABP increased with worsening albuminuria, whereas galectin-3 did not. In multivariate analyses, eGFR was the only independent predictor of all three biomarkers. **Conclusions**: In IgAN, galectin-3, MR-proANP, and H-FABP concentrations are closely linked to renal function and markers of cardiovascular remodeling. These findings suggest that galectin-3, MR-proANP, and H-FABP provide complementary information regarding the complex interaction between renal dysfunction and cardiovascular remodeling in IgA nephropathy. Prospective multicenter studies are warranted to determine their incremental prognostic value and potential clinical utility for cardiovascular risk stratification.

## 1. Introduction

IgA nephropathy (IgAN) is the most common primary glomerular disease worldwide, characterized by substantial clinical heterogeneity ranging from slowly progressive forms to rapidly deteriorating renal function [1,2]. The pathophysiology of IgAN is driven by mesangial IgA deposition and the subsequent inflammatory and fibrotic processes that contribute to chronic kidney damage [3]. In patients with chronic kidney disease (CKD), cardiovascular (CV) risk is markedly elevated, and CV mortality remains the leading cause of death [4].

In recent years, increasing emphasis has been placed on circulating biomarkers that may serve as early indicators of subclinical cardiovascular injury and renal deterioration. Galectin-3, a β-galactoside-binding lectin, has a molecular size of 31 kDa, plays a central role in fibrosis, inflammation and tissue remodeling; its circulating levels are elevated in CKD and have been associated with CV morbidity and mortality [5,6]. The lectin pathway of the complement system plays a key role in the development and progression of IgA nephropathy (IgAN). Among cardiac biomarkers, mid-regional proatrial natriuretic peptide (MR-proANP), which has a molecular size of 5.1 kDa, reflects the stress and volume overload of the myocardial wall, while myocardial-type fatty acid binding protein (H-FABP), which has a molecular size of 15 kDa, is considered a sensitive marker of early myocardial ischemic injury [7,8,9].

These biomarkers may therefore provide valuable insight into the interplay between CV remodeling, arterial stiffness and renal impairment in CKD patients.

The cardiovascular burden in IgA nephropathy is multifactorial and extends beyond traditional cardiovascular risk factors. Chronic inflammation, endothelial dysfunction, oxidative stress, vascular calcification, neurohormonal activation, and myocardial fibrosis all contribute to the development of subclinical cardiac remodeling during progressive renal impairment. Consequently, biomarkers reflecting different biological pathways may provide complementary information regarding the cardiorenal continuum in IgA nephropathy.

Despite their potential utility, studies evaluating the combined relationship of galectin-3, MR-proANP and H-FABP with renal function in IgAN remain limited. Because cardiovascular alterations often precede overt clinical symptoms, biomarkers capable of detecting subclinical CV damage and renal deterioration may have significant prognostic relevance.

Therefore, the present study aimed to investigate the relationship between three mechanistically distinct cardiovascular biomarkers—galectin-3, MR-proANP, and H-FABP—and renal function, echocardiographic characteristics, and arterial stiffness in patients with biopsy-proven IgA nephropathy [10]. We hypothesized that renal dysfunction represents the principal determinant of circulating biomarker concentrations and that these biomarkers provide complementary information regarding cardiovascular remodeling within the cardiorenal continuum.

## 2. Materials and Methods

### 2.1. Study Design and Population

This cross-sectional observational study included patients with biopsy-proven IgA nephropathy (IgAN) who were regularly followed at the nephrology outpatient clinic of our department. A total of 90 consecutive adult patients were examined, but 76 were enrolled in the study, due to sample collection or technical problems. All participants were clinically stable at the time of inclusion.

Fourteen initially recruited patients were excluded because serum samples were unavailable or unsuitable for biomarker analysis owing to technical issues related to sample collection, processing, storage, or laboratory measurements. These exclusions were unrelated to clinical outcomes.

The study protocol was approved by the Regional Research Ethics Committee of the University of Pécs Clinical Center (reference no. 9737-PTE 2024, 25 January 2024), and all patients provided written informed consent in accordance with the Declaration of Helsinki.

### 2.2. Clinical and Laboratory Assessment

Demographic data, medical history, and cardiovascular risk factors—including hypertension, diabetes mellitus, dyslipidemia, smoking status, obesity, and metabolic syndrome—were recorded at study entry. Body mass index (BMI) was calculated as weight divided by height squared (kg/m^2^).

Venous blood samples were obtained after an overnight fast. Routine laboratory parameters, including serum creatinine, albumin, lipid profile, uric acid, phosphate, calcium, and hemoglobin levels, were measured using standard automated methods. Renal function was assessed by estimating the glomerular filtration rate (eGFR) using the CKD-EPI formula. Patients were categorized into two groups according to renal function: CKD stages 1–2 (eGFR ≥ 60 mL/min/1.73 m^2^) and CKD stages 3–5 (eGFR < 60 mL/min/1.73 m^2^).

Urinary albumin excretion was assessed by urine albumin-to-creatinine ratio (UACR) and albuminuria measurements from spot urine samples.

### 2.3. Biomarker Measurements

Serum concentrations of galectin-3, mid-regional pro-atrial natriuretic peptide (MR-proANP), and heart-type fatty acid–binding protein (H-FABP) were measured using commercially available enzyme-linked immunosorbent assay (ELISA) kits (Thermo Fisher Scientific, Waltham, MA, USA, Shanghai Jinkang Bioengineering Co., Ltd., Shanghai, China and BT Lab, Shanghai, China) according to the manufacturers’ instructions. All samples were analyzed in duplicate by laboratory personnel blinded to clinical data. For the galectin-3 assay, the reported intra- and inter-assay coefficients of variation were 7.5% and 5.4%, respectively, while for the H-FABP assay they were 3.9% and 6.2%, respectively. Intra- and inter-assay precision data for the MR-proANP assay were not publicly available from the manufacturer.

### 2.4. Cardiovascular Assessment

Transthoracic echocardiography (Mindray Consona N6, Shenzhen China Mindray Bio-Electronics Co., Ltd., Shenzhen, China) was performed in all patients by experienced cardiologists according to contemporary recommendations of the American Society of Echocardiography and the European Association of Cardiovascular Imaging. Left ventricular dimensions, ejection fraction (EF), diastolic function parameters, and left ventricular mass index (LVMI) were assessed. Left ventricular hypertrophy was defined based on LVMI values. Left ventricular ejection fraction was assessed using the modified biplane Simpson method whenever feasible.

Transmitral flow was assessed using pulsed-wave Doppler imaging from the apical four-chamber view. Peak early (E) and late (A) diastolic transmitral velocities were measured, and the E/A ratio was calculated as an indicator of left ventricular diastolic filling. A comprehensive grading of diastolic dysfunction was not performed because tissue Doppler and left atrial pressure-related parameters were not available for all participants.

Arterial stiffness was evaluated using an oscillometric Arteriograph device. Pulse wave velocity of the aorta (PWVao), central systolic blood pressure (SBPao), diastolic area index (DAI), and other central hemodynamic parameters were recorded under standardized conditions.

### 2.5. Statistical Analysis

Continuous variables were evaluated for normality using the Shapiro–Wilk test. Data are presented as mean ± standard deviation (SD) for normally distributed variables, or as median with interquartile range (IQR) for non-normally distributed data. Categorical variables are expressed as frequencies and percentages.

To compare clinical characteristics and biomarker concentrations between patients with preserved (eGFR ≥ 60 mL/min/1.73 m^2^) and reduced (eGFR < 60 mL/min/1.73 m^2^) renal function, the independent samples *t*-test or the Mann–Whitney U test was employed, as appropriate. Differences between multiple groups (e.g., CKD stages) were assessed using one-way ANOVA or the Kruskal–Wallis test with post hoc Bonferroni correction. Categorical data were compared using the chi-square test or Fisher’s exact test.

Due to their skewed distribution, serum concentrations of galectin-3, MR-proANP, and H-FABP underwent logarithmic transformation before correlation and regression analyses. Bivariate associations were assessed using Pearson’s or Spearman’s correlation coefficients.

To identify independent determinants of each biomarker, multivariate linear regression models were constructed using a backward stepwise approach. Variables that reached a significance level of *p* < 0.10 in univariate analyses or possessed established clinical relevance (age, sex, BMI) were entered into the models. To ensure model validity, collinearity was assessed using the Variance Inflation Factor (VIF), with a cut-off value of <3. All statistical tests were two-sided, and a *p*-value <0.05 was considered statistically significant. Statistical analyses were performed using SPSS software (version 25.0 or higher).

Variables included in multivariable linear regression models were selected based on their clinical relevance and statistical significance in univariable analyses while avoiding model overfitting due to the relatively limited sample size.

## 3. Results

A total of 90 consecutive patients were screened. Fourteen patients were excluded because biomarker measurements could not be reliably performed, leaving 76 patients for the final analyses.

The cohort comprised 41 men (54%) and 35 women (46%), with a mean age of 56.6 ± 11.9 years. Hypertension was present in 80% of patients, metabolic syndrome in 49%, dyslipidemia in 38%, and obesity in 33%. Baseline demographic, laboratory, echocardiographic, arterial stiffness, and biomarker characteristics are shown in Table 1.

Based on renal function, patients were stratified into two groups: eGFR ≥ 60 mL/min/1.73 m^2^ (n = 36) and eGFR < 60 mL/min/1.73 m^2^ (n = 40). Patients with reduced eGFR were significantly older (59.9 ± 10.8 vs. 53.0 ± 12.3 years; *p* = 0.011) and had lower hemoglobin concentrations (*p* < 0.001). Serum phosphate, UACR, and albuminuria were significantly higher in the eGFR < 60 group (all *p* < 0.05) (Table 1).

Echocardiography demonstrated a modest but significant reduction in left ventricular ejection fraction in patients with eGFR < 60 mL/min/1.73 m^2^ (60.9 ± 6.2 vs. 64.2 ± 6.3%; *p* = 0.049). No significant differences were observed in left ventricular mass index, chamber dimensions, or diastolic function parameters between groups (Table 1).

Among arterial stiffness indices, pulse wave velocity of the aorta showed a non-significant trend across eGFR categories (*p* = 0.087), while other central hemodynamic parameters did not differ significantly (Table 1).

There were significantly higher galectin-3 levels in females (10.7 vs. 9.08; *p* = 0.037), but the MR-proBNP and H-FABP did not differ significantly between the genders.

**Figure 1:**
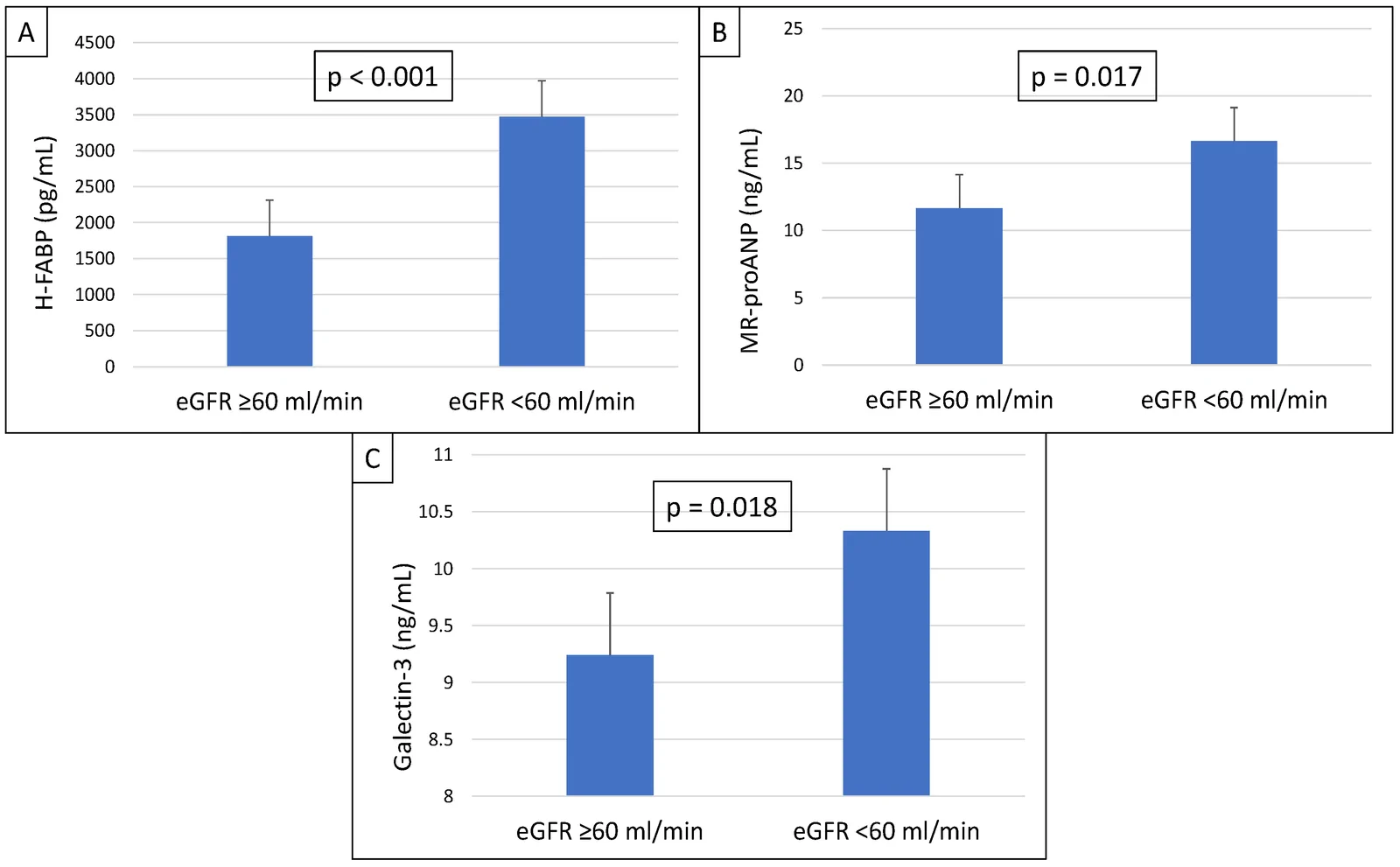
H-FABP (**A**), MR-proANP (**B**) and serum galectin-3 (**C**) levels in patients with CKD stages 1–2 and CKD stages 3–5.

Serum concentrations of all three investigated biomarkers increased significantly with declining renal function. Patients with eGFR < 60 mL/min/1.73 m^2^ exhibited higher galectin-3 levels (10.33 ± 2.32 vs. 9.24 ± 2.33 ng/mL; *p* = 0.011), higher MR-proANP concentrations (*p* < 0.001), and markedly elevated H-FABP levels (3469.6 ± 1548.0 vs. 1838.3 ± 978.8 pg/mL; *p* < 0.001) (Table 1, Figure 1).

**Figure 2:**
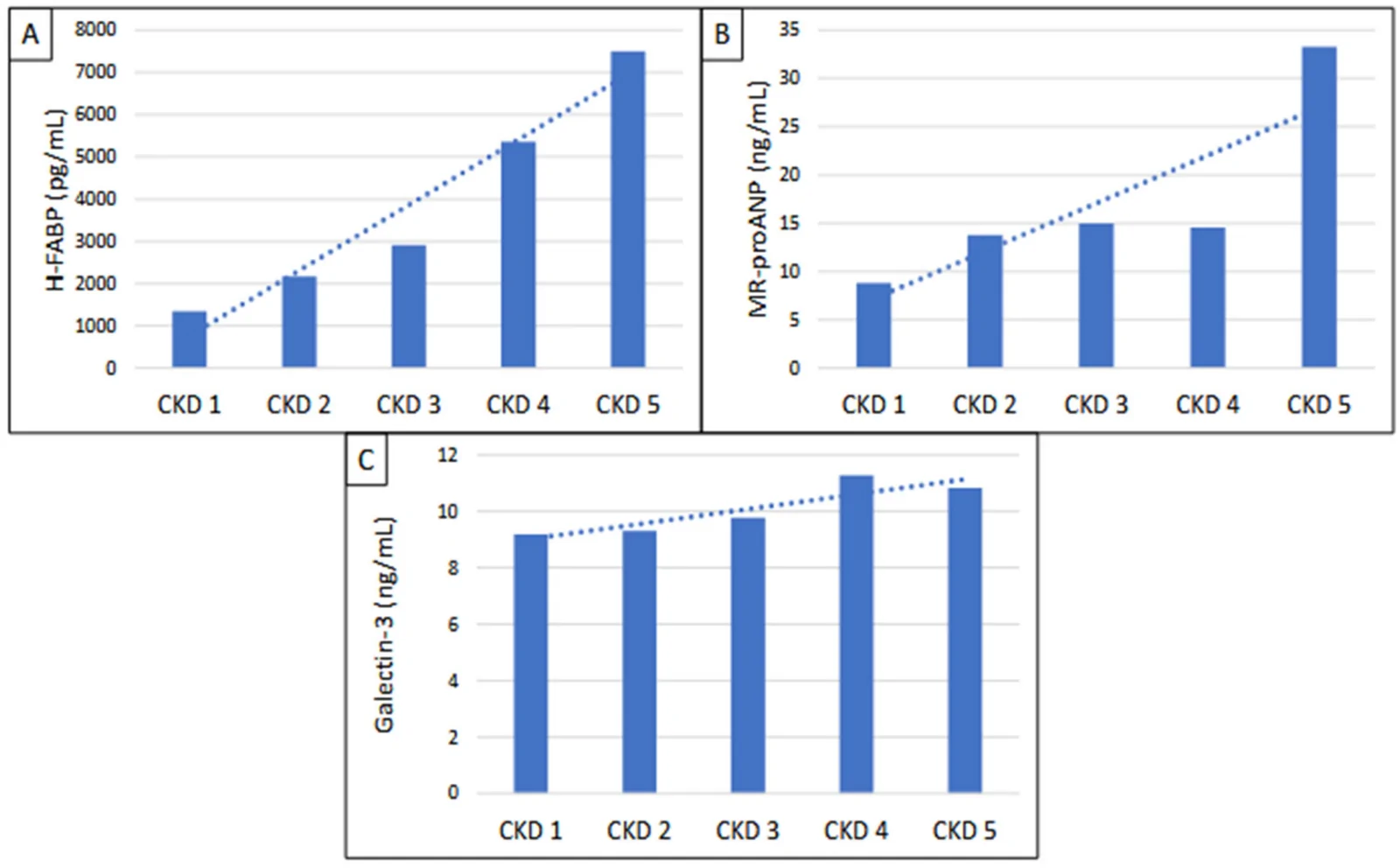
Trend analysis: H-FABP (**A**), MR-proANP (**B**) and serum galectin-3 (**C**) levels change in declining eGFR.

Trend analysis confirmed a progressive rise in galectin-3, MR-proANP, and H-FABP concentrations with worsening renal function (Figure 2).

Patients were further stratified according to the median values of galectin-3 (9.45 ng/mL), MR-proANP (8.78 ng/mL), and H-FABP (2538.91 pg/mL). Individuals in the high galectin-3 group had significantly higher MR-proANP and H-FABP concentrations compared with those in the low galectin-3 group (*p* = 0.01 and *p* < 0.001, respectively). Similarly, patients with high MR-proANP exhibited significantly elevated galectin-3 and H-FABP levels (both *p* < 0.001). In the high H-FABP group, both galectin-3 and MR-proANP concentrations were significantly increased (both *p* < 0.001) (Table 2).

Across all biomarker-based classifications, higher biomarker levels were consistently associated with significantly lower eGFR values (all *p* < 0.001) (Table 2).

**Figure 3:**
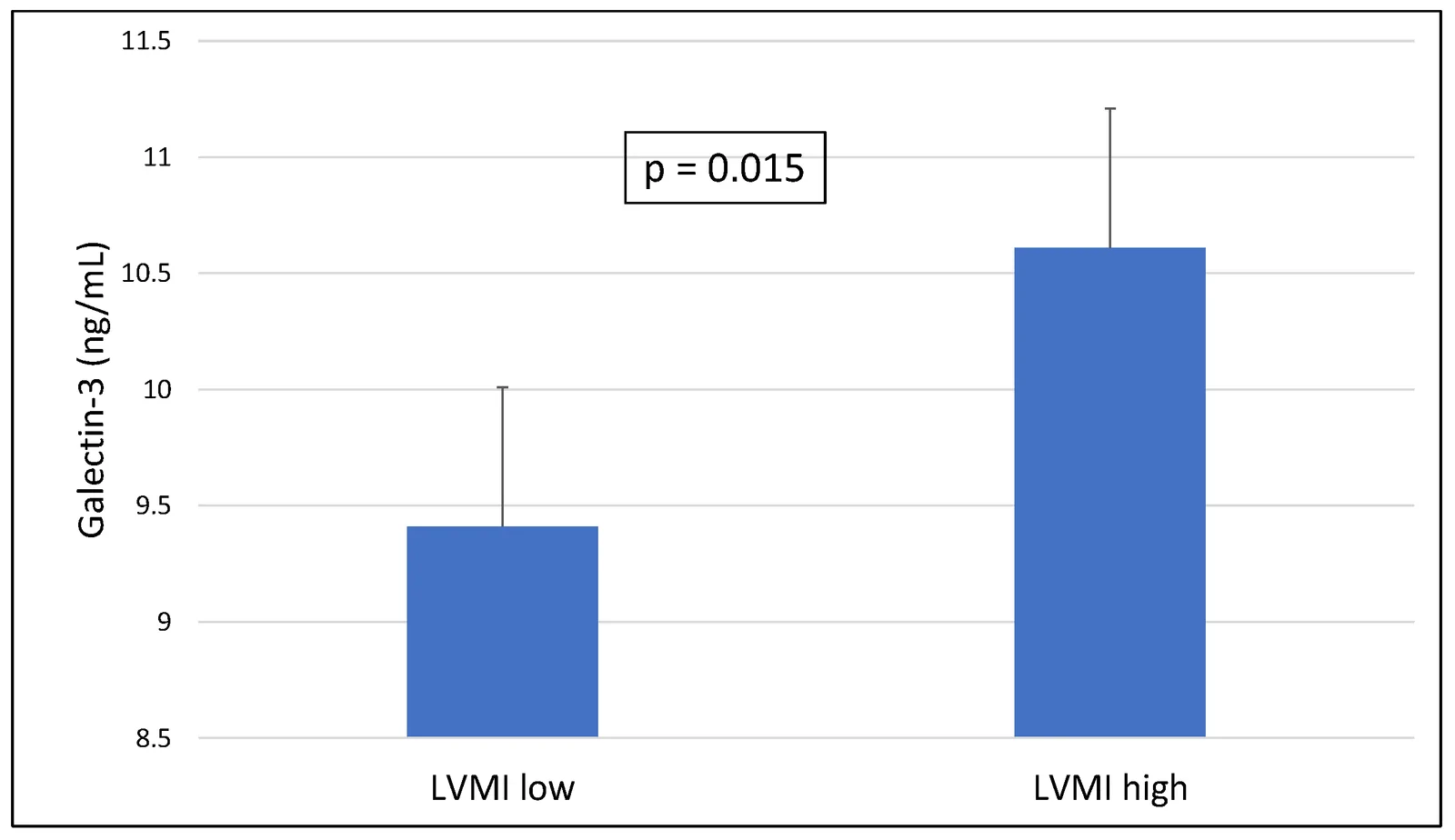
Serum galectin-3 levels difference in case of high and low LVMI.

When patients were stratified according to left ventricular mass index, serum galectin-3 concentrations were significantly higher in those with elevated LVMI (*p* = 0.015). In contrast, MR-proANP and H-FABP levels did not differ significantly between LVMI categories (Figure 3).

In Pearson correlation analyses, galectin-3 was significantly associated with age, serum albumin, pulse wave velocity of the aorta, and eGFR. MR-proANP concentrations correlated with HDL-cholesterol, uric acid, serum albumin, phosphate, eGFR, and microalbuminuria. H-FABP levels showed significant associations with age, body mass index, obesity, metabolic syndrome, phosphate, eGFR, and microalbuminuria (Table 3).

To identify independent determinants of biomarker concentrations, separate multivariate linear regression models were constructed for galectin-3, MR-proANP, and H-FABP after logarithmic transformation of biomarker values (Table 4).

In the multivariate model for galectin-3, the estimated glomerular filtration rate (eGFR) emerged as the only independent predictor (standardized β = −0.31, *p* = 0.002). Age and serum albumin showed borderline associations but did not retain statistical significance after adjustment. No echocardiographic or arterial stiffness parameter independently predicted galectin-3 concentrations.

In the model for MR-proANP, eGFR was again the strongest and only independent determinant (β = −0.39, *p* < 0.001). Associations with albuminuria, serum albumin, and central aortic systolic blood pressure observed in univariate analyses were attenuated and lost significance in the multivariate model.

Similarly, in the H-FABP model, eGFR remained the dominant independent predictor (β = −0.58, *p* < 0.001). Phosphate levels showed a weak independent association, while age, body mass index, and albuminuria did not reach statistical significance after adjustment.

Overall, across three distinct biomarkers reflecting fibrosis, myocardial wall stress, and subclinical myocardial injury, renal function—expressed by eGFR—consistently emerged as the principal independent determinant of biomarker concentrations, whereas cardiovascular structural and hemodynamic parameters did not retain independent associations after multivariable adjustment (Table 4).

### Central Aortic Pressure and Albuminuria Subgroup Analyses

**Figure 4:**
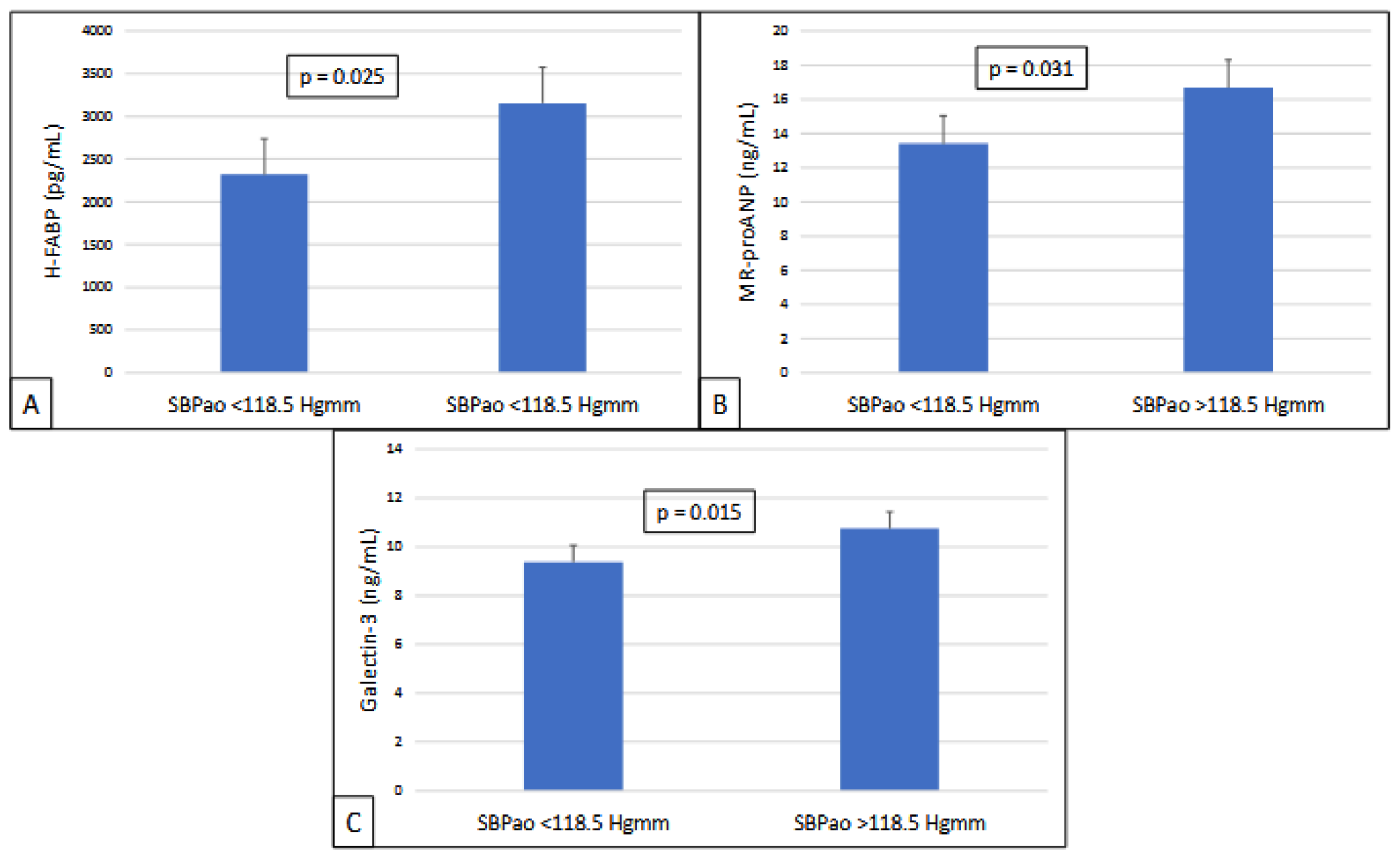
H-FABP (**A**), MR-proANP (**B**) and serum galectin-3 (**C**) level differences in cases of high and low SBPao.

Stratification by central aortic systolic blood pressure (SBPao; cut-off 118.5 mmHg) demonstrated significantly higher galectin-3, MR-proANP, and H-FABP levels in patients with elevated SBPao (Figure 4).

**Figure 5:**
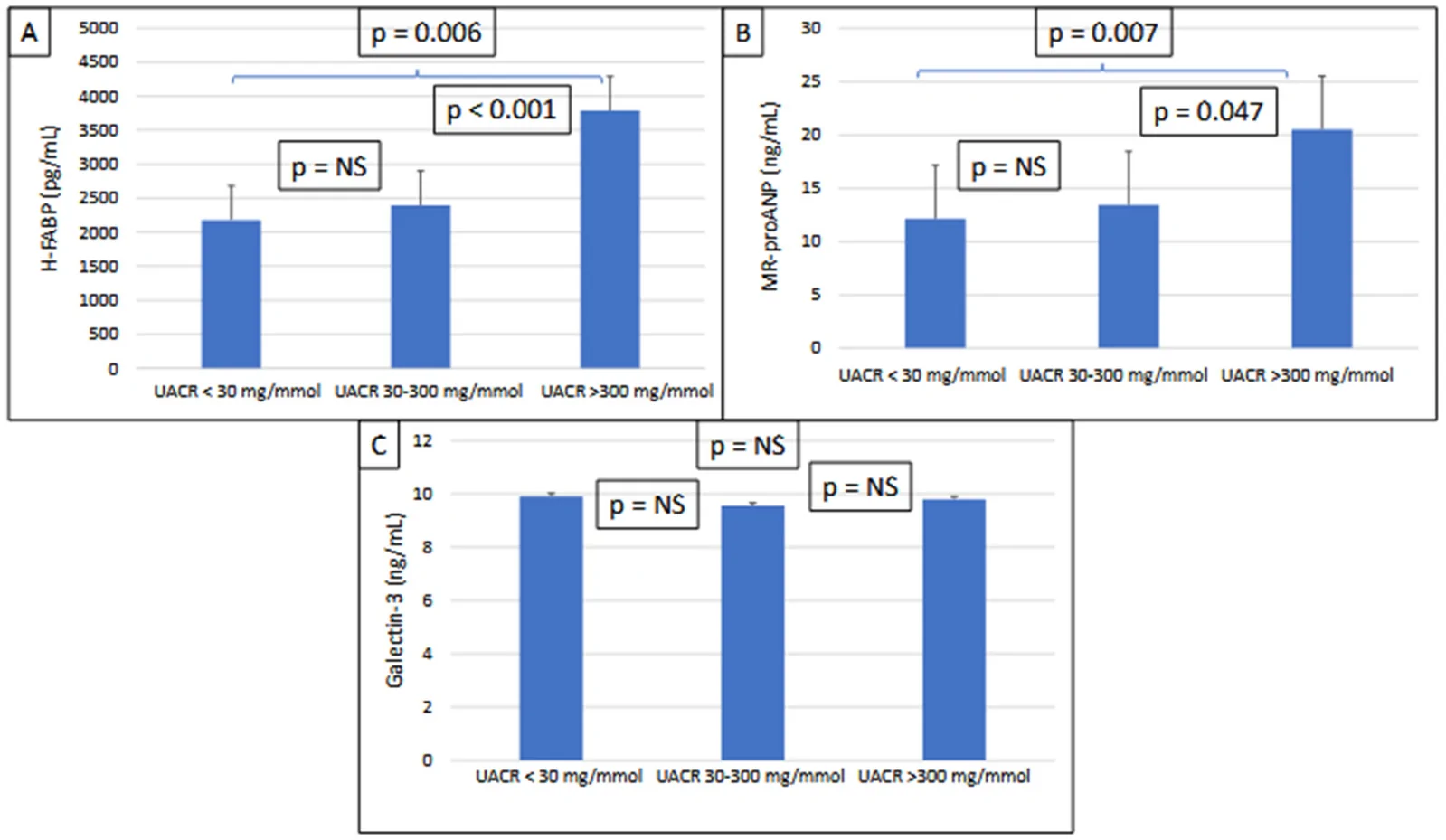
H-FABP (**A**), MR-proANP (**B**) and serum galectin-3 (**C**) levels in different groups of UACR (normal buminuria vs. microalbuminuria vs. macroalbuminuria).

When patients were categorized according to albuminuria severity (UACR < 30 mg/mmol, 30–300 mg/mmol, >300 mg/mmol), MR-proANP and H-FABP concentrations increased significantly with worsening albuminuria, whereas galectin-3 levels did not differ significantly between albuminuria categories (Figure 5).

## 4. Discussion

The present cross-sectional study investigated the relationship between three mechanistically distinct cardiovascular biomarkers—galectin-3, MR-proANP, and H-FABP—and renal function, echocardiographic characteristics, and arterial stiffness in patients with biopsy-proven IgA nephropathy. The principal finding of our study is that declining renal function, reflected by lower eGFR, represents the strongest independent determinant of all three biomarkers. Although each biomarker demonstrated significant associations with selected cardiovascular parameters in univariable analyses, these relationships were largely attenuated after adjustment for renal function, emphasizing the dominant influence of kidney dysfunction on circulating biomarker concentrations.

The interaction between the kidney and the cardiovascular system is increasingly recognized as a bidirectional and self-perpetuating process. Progressive nephron loss promotes chronic inflammation, oxidative stress, endothelial dysfunction, activation of the renin–angiotensin–aldosterone system, sympathetic overactivity, and accumulation of uremic toxins. Together, these processes contribute to myocardial fibrosis, left ventricular remodeling, vascular stiffening, and impaired myocardial relaxation. Conversely, cardiovascular dysfunction may further aggravate renal injury through reduced renal perfusion and persistent neurohormonal activation. This complex cardiorenal interaction provides a plausible biological explanation for the observed associations between renal function and circulating cardiovascular biomarkers.

Recent evidence further supports the concept that cardiovascular risk in CKD is governed by complex and often nonlinear interactions between renal dysfunction, metabolic abnormalities, and vascular injury. In particular, the relationship between serum uric acid and cardiovascular disease appears to be nonlinear, highlighting the multifactorial nature of cardiorenal risk stratification [11].

The strong and consistent inverse association between eGFR and biomarker concentrations likely reflects a combination of impaired renal clearance and the systemic CV alterations accompanying progressive chronic kidney disease. Declining kidney function is known to influence the circulating levels of numerous CV biomarkers, including natriuretic peptides and cardiac troponins, through reduced elimination as well as increased myocardial stress and inflammation. Our results extend these observations to galectin-3 and H-FABP in the specific context of IgA nephropathy.

Emerging evidence also indicates that epigenetic mechanisms contribute to progressive renal fibrosis and chronic inflammation, reinforcing the concept that renal dysfunction is driven by multiple interconnected molecular pathways extending beyond traditional hemodynamic mechanisms [12].

Importantly, CV structural and hemodynamic parameters, including LVMI, PWV, and SBPao, did not retain independent associations with biomarker concentrations after adjustment for renal function [13,14]. This suggests that the apparent relationships observed in univariate analyses are largely mediated by renal impairment rather than representing primary CV determinants. The finding underscores the dominant role of kidney dysfunction in shaping the circulating biomarker profile in IgAN.

Nevertheless, the observed associations between higher biomarker levels and adverse CV features—such as increased LVMI, elevated SBPao, and greater albuminuria—remain clinically relevant. These relationships likely reflect shared underlying mechanisms, including vascular stiffness, volume overload, and low-grade myocardial injury, which become increasingly prominent as renal function declines.

Taken together, our findings highlight the importance of interpreting CV biomarkers in IgA nephropathy within the context of renal function. While galectin-3, MR-proANP, and H-FABP may provide valuable insight into subclinical CV remodeling, their circulating concentrations are strongly modulated by kidney function, which must be accounted for in both clinical and research settings.

IgAN is the most prevalent primary glomerulopathy worldwide [1,2,3], and although traditionally considered a kidney-limited disease, it is now well established that patients with IgAN carry a heightened cardiovascular risk compared with the general population. Declines in kidney function strongly predict morbidity and mortality [4], and even modest decreases in eGFR have disproportionate effects on cardiovascular outcomes. As CKD progresses, a wide spectrum of cardiovascular abnormalities emerges, including arterial stiffness, increased central systolic pressure, left ventricular hypertrophy (LVH), diastolic dysfunction, and subclinical myocardial injury [1,15]. Accordingly, biomarkers that reflect these processes may provide clinicians with valuable information regarding disease severity, CV risk, and prognosis.

### 4.1. Galectin-3 and Its Relationship to Renal and Cardiovascular Alterations

Galectin-3, a β-galactoside-binding lectin, plays a pivotal role in tissue fibrosis, inflammation, macrophage activation, and extracellular matrix turnover [16,17,18]. Elevated galectin-3 levels have been consistently associated with adverse cardiovascular outcomes and heart failure progression [5,19], as well as with accelerated renal function decline [6,20]. In our cohort, galectin-3 concentrations were significantly higher in patients with reduced eGFR and in those with increased LVMI, suggesting the presence of myocardial remodeling in more advanced IgAN. These findings are in line with evidence that galectin-3 is upregulated in cardiac fibrotic tissue and marks activated macrophages in heart failure [19,20].

Furthermore, we observed correlations between galectin-3 and arterial stiffness indices, including PWVao. Prior studies have demonstrated that galectin-3 is associated with aortic stiffness and impaired vascular function [21,22], supporting the hypothesis that galectin-3 contributes to systemic fibrotic and vascular remodeling in CKD. Notably, although significant associations were observed with LVMI and PWVao, eGFR remained the only independent predictor of galectin-3 levels. This finding is compatible with earlier work showing that impaired renal clearance plays a major role in the accumulation of galectin-3 in CKD [23,24,25,26], and highlights the need for careful interpretation of galectin-3 levels in patients with declining kidney function. Despite the fact that galectin-3 is not eliminated renally, its levels still increase with renal function decline, which may indicate a profibrotic effect and may play a causal role in CV changes.

In our study, female IgAN patients had higher galectin-3 levels than male patients, suggesting a gender-specific biological basis. Since galectin-3 is associated with macrophage activation and tissue fibrosis, this persistent difference may indicate that the dynamics of inflammatory–fibrotic processes in female and male patients are slightly different, regardless of age or stage of proteinuria similarly in the recent literature data [27].

### 4.2. MR-proANP as a Marker of Myocardial Wall Stress and Volume Overload

MR-proANP is a stable fragment of the prohormone of atrial natriuretic peptide and reflects myocardial stretch, volume overload, and wall tension [7,28]. Although natriuretic peptides are widely used in the diagnosis and risk stratification of heart failure [29,30,31], their concentrations are influenced by renal function, blood pressure, and arterial stiffness—factors commonly deranged in CKD. In our study, MR-proANP levels were significantly higher in the lower-eGFR group, consistent with previous findings that kidney dysfunction reduces peptide clearance and enhances cardiac wall stress [32,33,34].

Moreover, MR-proANP correlated with biochemical markers such as phosphate, uric acid, albumin, and lipid parameters. These associations likely reflect the complex metabolic milieu characteristic of CKD, where fluid overload, endothelial dysfunction, mineral metabolism abnormalities, and oxidative stress collectively contribute to CV remodeling. Although MR-proANP levels tended to be higher in individuals with increased LVMI, the association did not remain significant after adjusting for renal function. This reinforces the notion that eGFR profoundly affects MR-proANP concentrations and may obscure more subtle cardiovascular associations. The concomitant elevation of MR-proANP in the high galectin-3 group suggests that fibrotic and inflammatory pathways may parallel myocardial wall stress in IgA nephropathy.

MR-proANP represents a stable surrogate marker of atrial natriuretic peptide release and reflects myocardial wall stress, intravascular volume expansion, and neurohormonal activation. Because chronic kidney disease is frequently accompanied by sodium retention, fluid overload, and increased cardiac preload, elevated MR-proANP concentrations may indicate both subclinical cardiovascular adaptation and reduced renal clearance. The observed association between MR-proANP and renal dysfunction therefore most likely reflects the integrated hemodynamic alterations characteristic of the cardiorenal syndrome.

### 4.3. H-FABP: A Sensitive Indicator of Subclinical Myocardial Injury

H-FABP is predominantly expressed in cardiomyocytes and is released rapidly into circulation following myocardial ischemia [9,35]. While initially validated as an early biomarker for acute coronary syndromes, H-FABP has emerged as a marker of chronic low-grade myocardial injury in CKD [36,37,38]. Our findings support this interpretation: H-FABP levels were significantly elevated in patients with reduced kidney function and correlated with BMI, metabolic syndrome, phosphate, proteinuria, and age.

These associations shed light on the multifaceted determinants of H-FABP in CKD. Proteinuria and metabolic syndrome both promote systemic inflammation, endothelial dysfunction, and microvascular rarefaction—mechanisms known to contribute to subclinical myocardial injury [39,40,41]. Several studies have reported that H-FABP levels are increased even in CKD patients without overt cardiac disease [40], suggesting that this biomarker may capture early cardiomyocyte stress long before clinical manifestations develop. Interestingly, in our multivariate analysis, eGFR was again the only independent determinant of H-FABP levels, emphasizing that impaired renal excretion plays a decisive role in circulating concentrations.

H-FABP is an established marker of myocardial injury that has attracted increasing attention as a potential indicator of subclinical cardiovascular damage in chronic kidney disease. Besides minor cardiomyocyte injury resulting from chronic pressure overload, microvascular dysfunction, and myocardial fibrosis, impaired renal elimination contributes substantially to elevated circulating H-FABP concentrations. The present findings therefore support the concept that H-FABP integrates information from both cardiac and renal pathophysiological pathways [42].

### 4.4. Renal Function as the Unifying Determinant of Biomarker Elevation

A major and noteworthy finding of our study is that across three mechanistically distinct biomarkers—representing fibrosis (galectin-3), myocardial stretch (MR-proANP) and myocardial injury (H-FABP)—renal function emerged as the only independent predictor. This observation aligns with accumulating evidence that eGFR profoundly influences biomarker levels in CKD, both through impaired renal clearance and via the systemic pathophysiologic environment associated with progressive nephron loss [43,44,45].

Renal dysfunction affects nearly every cardiovascular biomarker used in clinical practice, including troponins [46], natriuretic peptides [47], and inflammatory mediators. As a result, confounding by renal function is a persistent challenge in CV risk assessment in CKD populations. Nonetheless, this does not diminish the potential prognostic value of these biomarkers. Rather, it suggests that biomarker interpretation requires contextualization within kidney function strata. MR-proANP and H-FABP are low molecular weight peptides, the elevation of which suggests the role of the kidney. Their elevation may be a consequence of decreased renal function and decreased elimination, all of which may be less important from the renal perspective, but much more important from the heart perspective.

### 4.5. Implications for Cardiovascular Risk Stratification in IgAN

The consistent elevation of galectin-3, MR-proANP, and H-FABP in patients with more advanced CKD suggests that these biomarkers may be valuable in identifying IgAN patients at higher risk for subclinical cardiovascular disease. Biomarker-guided approaches have been proposed for CKD progression monitoring and CV risk prediction [48,49], and our findings extend this rationale to the IgAN population. Specifically, these biomarkers may help detect early myocardial fibrosis, ischemia, or wall stress before echocardiographic abnormalities become evident; support risk stratification among patients with preserved eGFR but high-risk phenotypes (e.g., proteinuria, hypertension, metabolic syndrome); aid in monitoring treatment response in interventions targeting CV or renal pathways.

The parallel increase in galectin-3 and MR-proANP may reflect the coexistence of fibrotic activity and myocardial wall stress in IgA nephropathy; however, this association appears to be strongly influenced by renal function. These elevated biomarker levels indicate not only a decline in kidney function, but also the activity of kidney disease.

Given the substantial cardiovascular morbidity observed in IgAN, integrating biomarker profiling with imaging and hemodynamic assessments could offer a more nuanced approach to cardiorenal risk management. Although modest reductions in left ventricular ejection fraction were observed among patients with more advanced renal impairment, systolic function remained largely preserved throughout the study population. This finding suggests that structural and biochemical alterations may precede clinically overt systolic dysfunction in IgA nephropathy. More sensitive imaging techniques, including speckle-tracking echocardiography and assessment of global longitudinal strain, may identify earlier stages of myocardial dysfunction and should be incorporated into future prospective investigations.

Increased arterial stiffness represents another important component of cardiovascular remodeling in chronic kidney disease. Progressive vascular calcification, endothelial dysfunction, collagen deposition, and reduced arterial compliance contribute to elevated pulse wave velocity, increased left ventricular afterload, and subsequent myocardial remodeling. The observed relationships between arterial stiffness and circulating biomarkers further support the concept that these biomarkers reflect systemic cardiorenal remodeling rather than isolated cardiac injury.

### 4.6. Clinical Implications

Although none of the investigated biomarkers can currently be recommended for routine clinical use in patients with IgA nephropathy, our findings suggest that galectin-3, MR-proANP, and H-FABP may provide complementary information regarding cardiovascular involvement beyond conventional clinical assessment. Combined evaluation of biomarkers reflecting fibrosis, myocardial wall stress, and myocardial injury may improve future cardiovascular risk stratification, particularly in patients with declining renal function. Nevertheless, prospective multicenter studies are required to establish clinically meaningful cut-off values, determine incremental prognostic value beyond established cardiovascular risk factors, and evaluate cost-effectiveness before implementation into routine nephrology practice.

Beyond structural remodeling, increasing evidence suggests that platelet activation and thrombo-inflammatory pathways contribute to cardiovascular complications in chronic kidney disease. Experimental data identifying NR4A1 as a key regulator of platelet activation further emphasize the complex biological mechanisms linking renal dysfunction with cardiovascular injury [50].

### 4.7. Strengths of the Study

This study has several important strengths. First, it included a well-characterized cohort of patients with biopsy-proven IgA nephropathy, thereby minimizing diagnostic heterogeneity. Second, three biologically distinct cardiovascular biomarkers reflecting myocardial fibrosis (galectin-3), myocardial wall stress (MR-proANP), and myocardial injury (H-FABP) were evaluated simultaneously within the same patient population. Third, circulating biomarkers were analyzed together with echocardiographic parameters and arterial stiffness, providing a comprehensive assessment of cardiorenal remodeling. Finally, multivariable analyses enabled evaluation of the independent contribution of renal function to biomarker concentrations while accounting for major cardiovascular variables.

### 4.8. Study Limitations

This study has several limitations. First, its cross-sectional design precludes causal inference and limits interpretation to observed associations. Second, the relatively small sample size from a single tertiary referral center may reduce statistical power and limit generalizability. Third, biomarkers were measured at a single time point; therefore, temporal changes and prognostic implications could not be evaluated. Fourth, advanced echocardiographic parameters, including global longitudinal strain and comprehensive diastolic function assessment, were unavailable for all participants. Fifth, residual confounding by medication use, inflammatory status, lifestyle factors, and other unmeasured variables cannot be completely excluded. Finally, external validation in independent multicenter cohorts is required before these findings can be translated into clinical practice.

## 5. Conclusions

In conclusion, renal function represents the principal determinant of circulating galectin-3, MR-proANP, and H-FABP concentrations in patients with IgA nephropathy. These biomarkers appear to reflect the complex interplay between renal dysfunction, cardiovascular remodeling, and arterial stiffness rather than isolated cardiac injury. Future prospective multicenter studies integrating serial biomarker measurements with advanced cardiovascular imaging are warranted to determine their prognostic significance and potential role in personalized cardiovascular risk assessment.

## Figures and Tables

**Table 1:** Baseline clinical data.

Clinical Parameters	Total IgAN Patients (n = 76)	IgAN Patients eGFR ≥ 60 mL/min (n = 36)	IgAN Patients eGFR < 60 min/min (n = 40)	*p*
Age (years)	56.61 ± 11.93	52.97 ± 12.26	59.88 ± 10.75	**0.011**
Gender (M/F)	41/35 (54/46)	22/14 (61/39)	19/21 (47/53)	0.338
HT (n/%)	61 (80)	28 (78)	33 (82)	0.820
DM (n/%)	14 (18)	7 (19)	7 (17)	1.000
Dyslipidemia (n)	29 (38)	13 (36)	16 (40)	0.911
Obesity (n/%)	25 (33)	11 (30)	14 (35)	0.867
BMI (kg/m^2^)	29.39 ± 6.08	28.31 ± 5.37	30.27 ± 6.55	0.249
Metabolic syndrome (n/%)	37 (49)	14 (39)	23 (57)	0.164
Smoking (n/%)	3 (4)	0 (0)	3 (7)	0.242
Kidney disease vintage (years)	26.50 ± 15.10	26.41 ± 13.94	26.58 ± 16.45	0.974
Laboratory results				
Total cholesterol (mmol/L)	5.05 ± 1.15	4.86 ± 1.01	5.21 ± 1.26	0.207
LDL cholesterol (mmol/L)	9.07 ± 50.56	15.91 ± 74.16	3.13 ± 1.13	0.760
HDL cholesterol (mmol/L)	1.34 ± 0.40	1.34 ± 0.28	1.34 ± 0.48	0.518
Triglycerides (mmol/L)	2.12 ± 2.21	1.83 ± 0.88	2.38 ± 2.91	0.840
Uric acid	290.76 ± 76.25	276.67 ± 67.48	304.44 ± 82.59	0.136
LDH (IU/L)	178.96 ± 46.39	183.39 ± 26.02	172.82 ± 65.96	0.575
Serum albumin (g/L)	44.33 ± 3.00	44.91 ± 2.92	43.73 ± 3.01	0.099
Hemoglobin (g/L)	143.67 ± 17.51	150.77 ± 13.95	137.13 ± 18.06	**<0.001**
Platelet count (G/L)	245.07 ± 60.47	240.92 ± 62.91	249.11 ± 58.59	0.567
Calcium (mmol/L)	2.42 ± 0.10	2.41 ± 0.07	2.43 ± 0.12	0.603
Phosphate (mmol/L)	1.10 ± 0.29	1.01 ± 0.17	1.18 ± 0.34	**0.017**
Echocardiography parameters				
LVEF (%)	62.50 ± 6.42	64.21 ± 6.29	60.90 ± 6.22	**0.049**
LVEDD (mm)	48.52 ± 3.81	48.45 ± 4.08	48.57 ± 3.68	0.916
LVESD (mm)	30.38 ± 8.13	28.72 ± 4.28	31.73 ± 10.18	0.848
IVSD (mm)	11.35 ± 1.11	11.43 ± 1.29	11.29 ± 0.98	0.564
E/A	1.01 ± 0.37	1.03 ± 0.36	1.00 ± 0.39	0.638
LVMI (g/m^2^)	108.04 ± 19.89	107.12 ± 21.68	108.73 ± 18.82	0.787
Arterial stiffness parameters				
PWVao (m/s)	10.57 (9.40–11.97)	10.15 (9.08–11.29)	11.30 (10.12–12.23)	0.087
SBPao (mmHg)	122.95 ± 22.57	122.50 ± 22.37	123.34 ± 23.15	0.459
DAI (%)	52.94 ± 4.91	53.62 ± 5.68	52.37 ± 4.18	0.395
cfPWV (m/s)	9.64 ± 1.99	9.39 ± 1.91	9.82 ± 2.07	0.472
Aortic pressure (mmHg)	114.35 ± 13.66	113.00 ± 14.45	115.30 ± 13.27	0.587
Kidney parameters				
eGFR (mL/min/1.73 m^2^)	57.74 ± 25.78	80.81 ± 9.82	36.98 ± 16.07	**<0.001**
UACR (mg/mmol)	94.47 ± 152.91	37.13 ± 59.20	153.71 ± 193.92	**<0.001**
Albuminuria (mg/L)	356.64 ± 763.71	120.03 ± 209.69	593.26 ± 1012.80	**0.007**
Biomarkers				
Galectin-3 (ng/mL)	9.81 ± 2.37	9.24 ± 2.33	10.33 ± 2.32	**0.011**
MR-proANP (ng/mL)	14.18 ± 10.49	11.51 ± 11.29	16.65 ± 9.17	**<0.001**
-FABP (pg/mL)	2604.51 ± 1510.07	1838.25 ± 978.83	3469.64 ± 1548.04	**<0.001**

HT: hypertension; DM: diabetes mellitus; BMI: body mass index; LDL: low-density lipoprotein; LDH: lactate dehydrogenase enzyme; LVEF: left ventricular ejection fraction; LVEDD: left ventricular end-diastolic diameter; LVESD: left ventricular end-systolic diameter; IVSD: interventricular septum diameter; E/A: early and late mitral inflow; LVMI: left ventricular mass index; PWVao: pulse wave velocity; SBPao: systolic blood pressure DAI: diastolic area index; cfPWV: carotid–femoral pulse wave velocity; eGFR: estimated glomerular filtration rate; UACR: urine albumin creatinine ratio; and MR-proANP: mid-regional prohormone of atrial natriuretic peptide. Bold: statistically significant.

**Table 2:** Clinical, laboratory, and echocardiographic characteristics according to biomarker categories.

	Galectin-3 (Cut-Off: 9.45)			MR-proANP (Cut-Off: 8.78)			H-FABP (Cut-Off: 2538.91)		
	Low Galectin-3 (n = 40)	High Galectin-3 (n = 45)	*p*	Low MR-proANP (n = 24)	High MR-proANP (n = 61)	*p*	Low H-FABP (n = 41)	High H-FABP (n = 44)	*p*
Female/Male	27/13	11/34	**<0.001**	23/1	15/46	**<0.001**	16/25	22/23	0.41
Age (years)	54.38 ± 12.42	58.58 ± 11.32	**0.048**	54.33 ± 13.18	57.24 ± 11.49	0.21	52.98 ± 12.17	60.70 ± 10.22	**0.003**
BMI (kg/m^2^)	28.29 ± 5.58	30.38 ± 6.28	0.09	27.52 ± 6.28	29.78 ± 5.69	0.10	27.30 ± 4.65	31.12 ± 6.81	**0.01**
UACR (mg/g)	44.74 ± 68.95	140.12 ± 196.72	**0.01**	84.91 ± 197.87	88.17 ± 129.21	0.88	53.28 ± 151.12	85.08 ± 95.64	0.27
MAU (mg/24 h)	249.57 ± 543.67	453.14 ± 933.64	**0.03**	139.02 ± 236.80	351.80 ± 379.69	0.06	101.92 ± 188.30	426.46 ± 706.41	**0.04**
LDL cholesterol (mmol/L)	3.03 ± 1.23	3.14 ± 1.08	0.72	3.09 ± 1.05	3.03 ± 1.23	0.74	3.07 ± 1.07	3.14 ± 1.10	0.78
HDL cholesterol (mmol/L)	1.37 ± 0.41	1.31 ± 0.39	0.46	1.43 ± 0.36	1.31 ± 0.41	0.18	1.44 ± 0.39	1.33 ± 0.40	0.22
Triglycerides (mmol/L)	1.84 ± 1.13	2.41 ± 2.93	0.18	1.79 ± 0.77	2.27 ± 2.56	0.22	1.75 ± 1.09	1.91 ± 1.25	0.46
Uric acid (µmol/L)	294.0 ± 73.0	287.0 ± 79.2	0.63	267.95 ± 58.80	301.94 ± 79.67	**0.04**	275.24 ± 53.25	343.53 ± 87.36	**<0.001**
Serum albumin (g/L)	45.43 ± 2.69	43.15 ± 2.88	**0.002**	45.06 ± 2.88	44.11 ± 2.99	0.09	45.23 ± 2.69	43.84 ± 2.91	**0.01**
Hb (g/L)	148.9 ± 14.8	139.7 ± 18.6	**0.01**	142.83 ± 12.21	145.56 ± 18.62	0.48	148.42 ± 14.35	143.88 ± 15.16	0.12
IVSd (mm)	11.07 ± 2.41	11.71 ± 1.44	0.12	10.11 ± 2.64	11.98 ± 1.27	**0.004**	11.06 ± 2.16	11.69 ± 1.89	0.15
LVEF (%)	64.23 ± 7.43	61.19 ± 5.23	0.06	63.84 ± 5.84	62.39 ± 6.75	0.29	63.60 ± 5.65	63.22 ± 5.91	0.78
LVMI (g/m^2^)	102.8 ± 20.0	111.8 ± 23.7	**0.03**	96.81 ± 21.38	109.82 ± 22.11	**0.01**	112.57 ± 26.26	102.72 ± 18.00	**0.04**
NT-proBNP (pg/mL)	121.0 ± 104.8	162.2 ± 226.2	0.48	87.00 ± 68.32	160.33 ± 195.24	**0.02**	75.16 ± 14.98	160.06 ± 237.79	**0.036**
eGFR (mL/min/1.73 m^2^)	67.6 ± 21.3	47.8 ± 26.3	**<0.001**	76.86 ± 18.18	50.74 ± 24.71	**<0.001**	75.16 ± 14.98	45.85 ± 20.09	**<0.001**
H-FABP (pg/mL)	2226 ± 1302	2933 ± 1678	**<0.001**	1453.63 ± 522.80	3093.61 ± 1550.48	**<0.001**	n.a.	n.a.	-
Galectin-3 (ng/mL)	n.a.	n.a.	-	9.29 ± 2.56	9.96 ± 2.23	**<0.001**	9.02 ± 2.03	10.37 ± 2.35	**<0.001**
MR-proANP (ng/mL)	11.34 ± 6.16	16.99 ± 13.17	**0.01**	n.a.	n.a.	-	9.65 ± 4.84	14.46 ± 7.49	**<0.001**

MR-proANP: mid-regional prohormon of atrial natriuretic peptide; H-FABP: heart-type fatty acid binding protein; BMI: body mass index; UACR: urine albumin-to-creatinine ratio; MAU: microalbuminuria; LDL: low-density liupoprotein; HDL: high-density lipoprotein; Hb: hemoglobin; IVSd: interventricular septum diameter; LVEF: left ventricular ejection fraction, LVMI: left ventricular mass index; NT-proBNP: N-terminal prohormone of brain natriouretic peptide; and eGFR: estimated glomerular filtration rate. Bold: statistically significant.

**Table 3:** Correlations between galectin-3, MR-proANP and H-FABP and selected clinical, laboratory, echocardiographic and arterial stiffness parameters.

	Galectin-3		MR-proANP		H-FABP	
	r	*p*	r	*p*	r	*p*
Age (years)	0.52	**0.005**	0.17	0.136	0.29	**0.016**
BMI (kg/m^2^)	0.20	0.360	0.06	0.619	0.35	**0.007**
Obesity	0.09	0.682	0.06	0.632	0.32	**0.016**
MetS	0.20	0.323	0.18	0.122	0.33	**0.006**
Serum albumin (g/L)	0.30	**0.009**	0.26	**0.029**	0.25	0.054
P (mmol/L)	0.09	0.492	0.39	**0.001**	0.40	**0.002**
PWVao (m/s)	0.28	**0.046**	0.12	0.391	0.06	0.722
LVMI (g/m^2^)	0.11	0.456	0.20	0.166	0.10	0.509
eGFR (mL/min/1.73 m^2^)	**−0.29**	**0.011**	**−0.38**	**0.001**	**−0.60**	**<0.001**
UACR (mg/mmol)	0.05	0.567	0.24	0.065	0.16	0.243

Correlations were assessed using Pearson’s or Spearman’s correlation coefficients, as appropriate. MR-proANP: mid-regional prohormone of atrial natriuretic peptide; H-FABP: heart-type fatty acid binding protein; BMI: body mass index; MetS: metabolic syndrome; P: phosphate; PWVao: aortic pulse wave velocity; LVMI: left ventricular mass index; eGFR: estimated glomerular filtration rate; and UACR: urine albumin-to-creatinine ratio. Bold: statistically significant.

**Table 4:** Multivariate linear regression analyses identifying independent determinants of galectin-3, MR-proANP and H-FABP.

**Dependent Variable: Galectin-3 (log-transformed)**			
**Independent variable**	**β (standardized)**	**95% CI**	***p***
Age (years)	0.18	−0.01 to 0.36	0.064
Sex (male)	−0.09	−0.27 to 0.10	0.332
**eGFR (mL/min/1.73 m^2^)**	**−0.31**	**−0.49 to −0.13**	**0.002**
Serum albumin (g/L)	0.15	−0.03 to 0.32	0.098
PWVao (m/s)	0.11	−0.05 to 0.28	0.176
LVMI (g/m^2^)	0.08	−0.07 to 0.24	0.301
**Dependent variable: MR-proANP (log-transformed)**			
**Independent variable**	**β (standardized)**	**95% CI**	***p***
Age (years)	0.10	−0.08 to 0.29	0.271
Sex (male)	−0.12	−0.30 to 0.06	0.184
eGFR (mL/min/1.73 m^2^)	**−0.39**	**−0.56 to −0.21**	**<0.001**
Serum albumin (g/L)	0.17	−0.01 to 0.35	0.061
UACR (mg/mmol)	0.14	−0.04 to 0.32	0.126
SBPao (mmHg)	0.09	−0.06 to 0.24	0.241
**Dependent variable: H-FABP (log-transformed)**			
**Independent variable**	**β (standardized)**	**95% CI**	***p***
Age (years)	0.16	−0.02 to 0.34	0.081
Sex (male)	0.07	−0.11 to 0.25	0.446
eGFR (mL/min/1.73 m^2^)	**−0.58**	**−0.72 to −0.43**	**<0.001**
BMI (kg/m^2^)	0.13	−0.04 to 0.29	0.132
P (mmol/L)	0.19	0.02 to 0.36	0.029
UACR (mg/mmol)	0.15	−0.03 to 0.33	0.101

Multivariate linear regression models are constructed separately for each biomarker. Biomarker concentrations are log-transformed prior to analysis. Standardized β coefficients are shown. Only variables with *p* < 0.10 in univariate analyses and clinical relevance are included to avoid overfitting. MR-proANP: mid-regional prohormone of atrial natriuretic peptide; H-FABP: heart-type fatty acid binding protein; BMI: body mass index; P: phosphate; PWVao: aortic pulse wave velocity; SBPao: aortic blood pressure; LVMI: left ventricular mass index; eGFR: estimated glomerular filtration rate; and UACR: urine albumin-to-creatinine ratio. Bold: statistically significant.

## Data Availability

The data underlying this article cannot be shared publicly due to Hungarian regulations and the privacy of individuals who participated in the study. The data could be shared upon reasonable request to the corresponding author if accepted by the Regional Committee for Medical and Health Research Ethics and local Data Protection Official.
